# Supplementary material for: An Approach to Design Adaptive Clinical Trials With Time‐to‐Event Outcomes Based on a General Bayesian Posterior Distribution
Source: Stat Med. 2025 Oct 9;44(23-24):e70207. doi: 10.1002/sim.70207 (PMC12510400; doi:10.1002/sim.70207)
Supplement: Supplementary file 2 — Figures S1–S9. Supporting Information. [file SIM-44-0-s002.pdf]

Supplementary material: An approach to design adaptive clinical trials with time-to-event outcomes based on the general Bayesian posterior distribution

James M. McGree[1,*], Antony M. Overstall[2], Mark Jones[3] and Robert K. Mahar[4,5]

[*]Corresponding author (email: james.mcgree@qut.edu.au)

[1]School of Mathematical Sciences
Queensland University of Technology
Brisbane, Australia

[2]School of Mathematical Sciences
University of Southampton
Southampton, United Kingdom

[3]Sydney School of Public Health
University of Sydney
Sydney, Australia

[4]School of Population and Global Health
University of Melbourne
Melbourne, Australia

[5]Clinical Epidemiology and Biostatistics Unit
Murdoch Children's Research Institute
Parkville, Australia

August 22, 2025

1 Algorithm for futility decision rule

Algorithm SM.1 describes the approach used to approximate the decision rule for futility at each interim analysis.

Algorithm SM.1 Approximation of decision rule for futility

Input $\boldsymbol{y}_{-r_k}, \boldsymbol{y}^*_{r_k}, \boldsymbol{x}_{1:n_k}, N_{\max}$
for $b = 1 : B$ **do**
  $\boldsymbol{v}_b \sim p(\boldsymbol{v})$ Simulate $N_{\max} - n_k$ treatment allocations
  $\boldsymbol{w}_b \sim p(\boldsymbol{w}|\boldsymbol{y}_{-r_k}, \boldsymbol{y}^*_{r_k}, \boldsymbol{x}_{1:n_k}, \boldsymbol{v}_b)$ Simulate data for participants who have not yet enrolled
  $\boldsymbol{z}_b \sim p(\boldsymbol{z}|\boldsymbol{y}_{-r_k}, \boldsymbol{y}^*_{r_k}, \boldsymbol{x}_{1:n_k})$ Simulate data for participants censored due to timing of interim
  Find $p(\beta|\boldsymbol{y}_{-r_k}, \boldsymbol{z}_b, \boldsymbol{w}_b, \boldsymbol{x}_{1:n_k}, \boldsymbol{v}_b)$ The updated posterior distribution of treatment effect
  $\boldsymbol{\Lambda}_b = \mathcal{I}(P(\beta < 0|\boldsymbol{y}_{-r_k}, \boldsymbol{z}_b, \boldsymbol{w}_b, \boldsymbol{x}_{1:n_k}, \boldsymbol{v}_b) > \Delta)$ Evaluate if decision rule would be triggered
end for
$\delta_f = \frac{1}{B}\sum_{b=1}^{B} \boldsymbol{\Lambda}_b$
if $\delta_f < \Delta_f$ **then**
  Stop the trial
end if

2 Additional results for the illustrative example

2.1 Power and Type I error results under vague priors for the super model

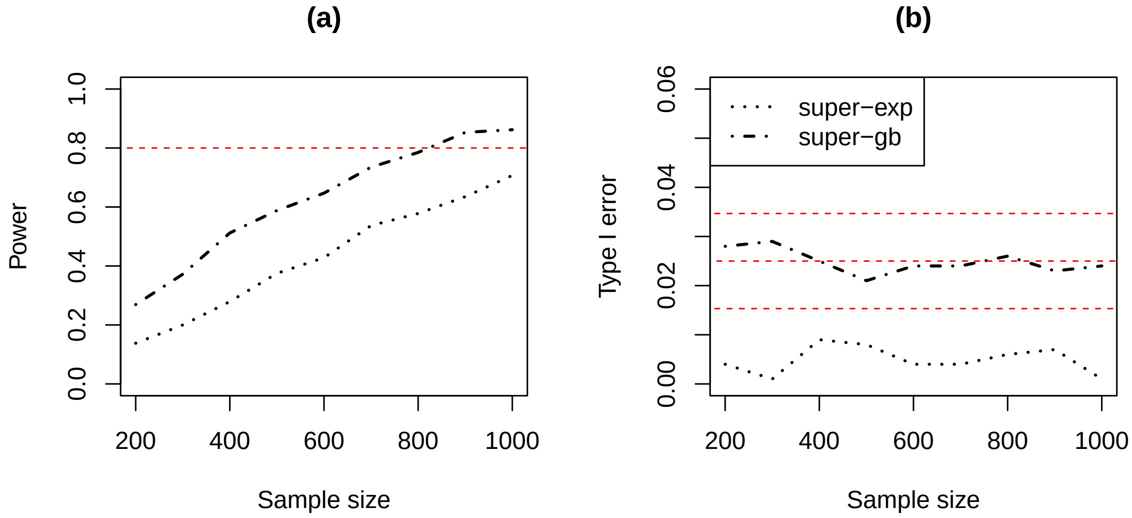

Figure SM.1: Plots of (a) Power and (b) Type I error for declaring the treatment as effective when vague prior information was assumed for the super model in the illustrative example when the exponential ('exp') and general Bayesian ('gb') models were used to estimate the treatment effect. All results are based on 1,000 trial simulations.

3 Additional results for the behaviour of the general posterior for increasing N

We explored the behaviour of the generalised posterior distribution formed via the partial likelihood as N increased. To do so, we simulated data as described in Section 7 of the main paper i.e. from the exponential, Weibull and super models under a range of treatment effects with different sample sizes. For each simulated data set, the posterior mode of the treatment effect was recorded. The results from this simulation are shown in Figures SM.2 and SM.3 for data generated from the exponential and Weibull model, respectively. As can be seen, across all sample sizes, the mean of the posterior modes was near the assumed value suggesting the approach is relatively unbiased. As the sample size increased, the variation in the posterior modes decreased suggesting the approach is concentrating around the assumed value of the treatment effect.

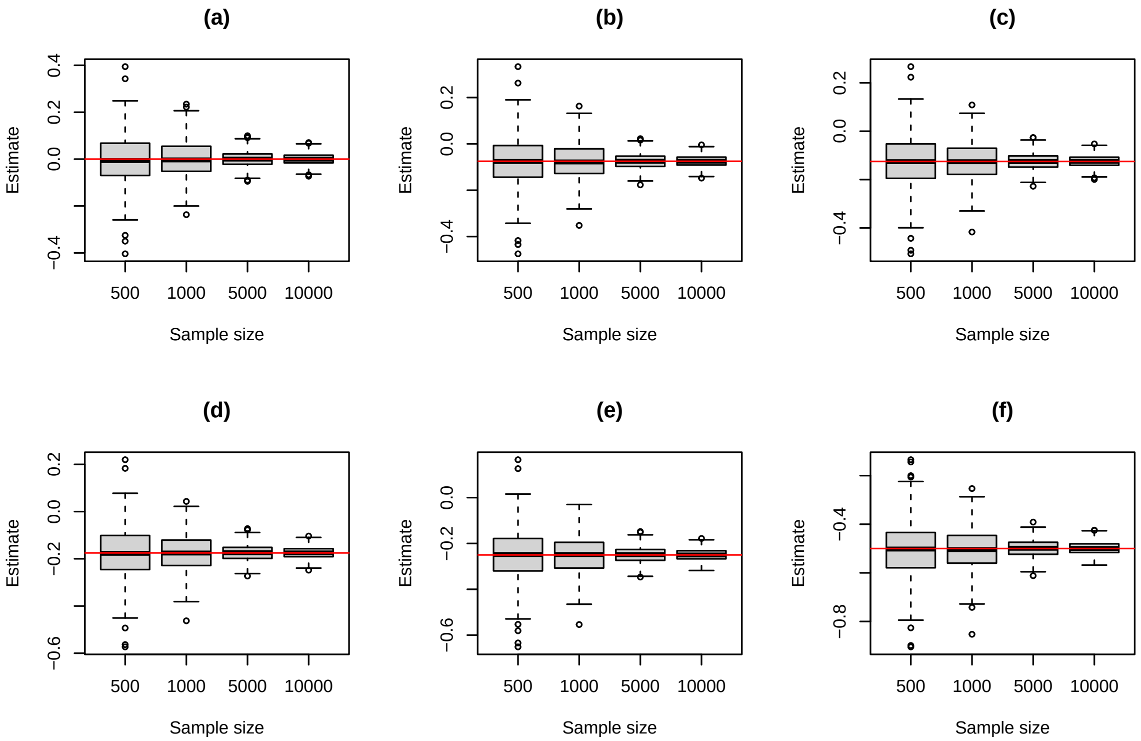

Figure SM.2: Distribution of posterior modes for the treatment effect under different sample sizes when data were generated from the exponential model with (a) $\beta = 0$, (b) $\beta = -0.075$, (c) $\beta = -0.125$, (d) $\beta = -0.175$, (e) $\beta = -0.25$, (f) $\beta = -0.5$ and the treatment effect was estimated based on the general posterior. All results are based on 500 trial simulations.

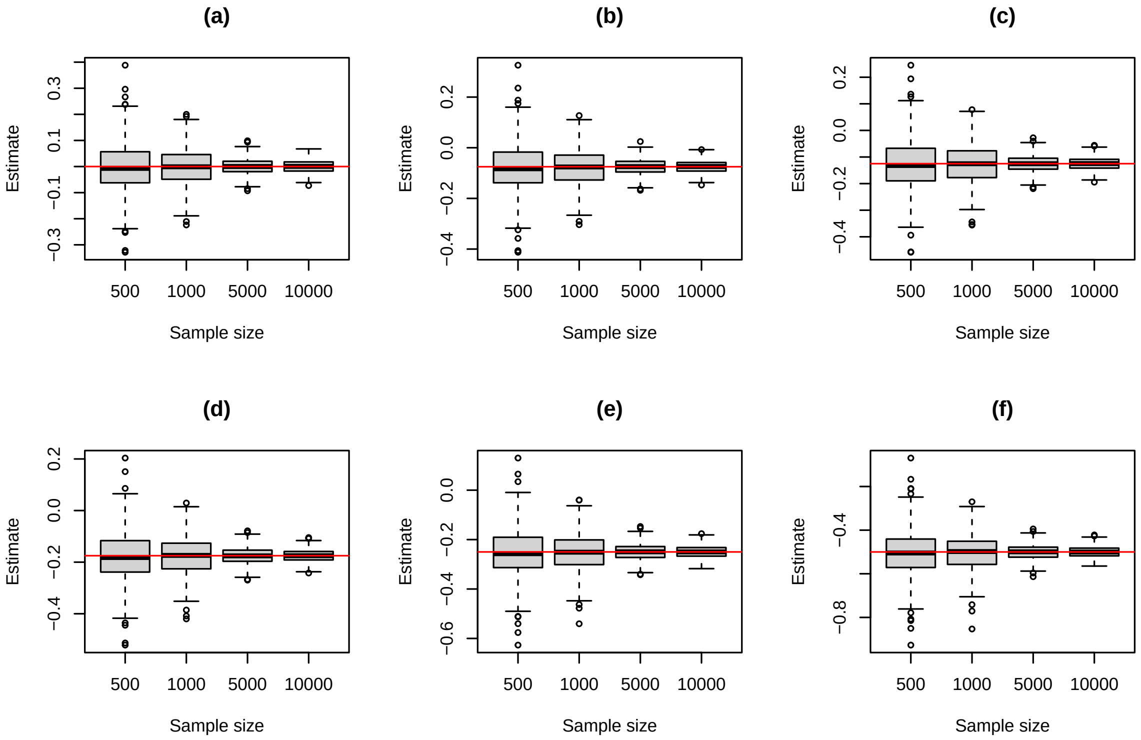

Figure SM.3: Distribution of posterior modes for the treatment effect under different sample sizes when data were generated from the Weibull model with (a) $\beta = 0$, (b) $\beta = -0.075$, (c) $\beta = -0.125$, (d) $\beta = -0.175$, (e) $\beta = -0.25$, (f) $\beta = -0.5$ and the treatment effect was estimated based on the general posterior. All results are based on 500 trial simulations.

4 Additional results for motivating clinical trial

4.1 Distribution of posterior modes for the treatment effect from trial simulation with a fixed sample size

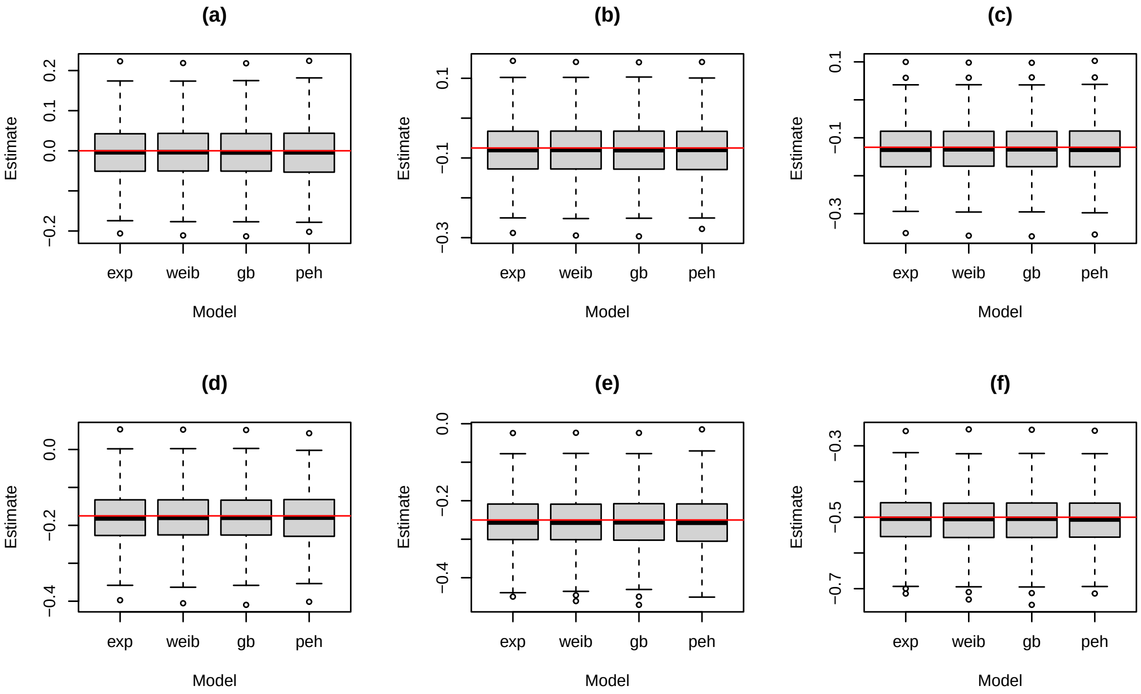

Figure SM.4: Distribution of posterior modes for the treatment effect from trial simulation with a fixed sample size when data were generated from the exponential model with (a) $\beta = 0$, (b) $\beta = -0.075$, (c) $\beta = -0.125$, (d) $\beta = -0.175$, (e) $\beta = -0.25$, (f) $\beta = -0.5$ and the treatment effect was estimated based on the exponential ('exp'), Weibull ('weib'), general Bayesian ('gb') and piecewise exponential hazard ('peh') models. All results are based on 500 trial simulations.

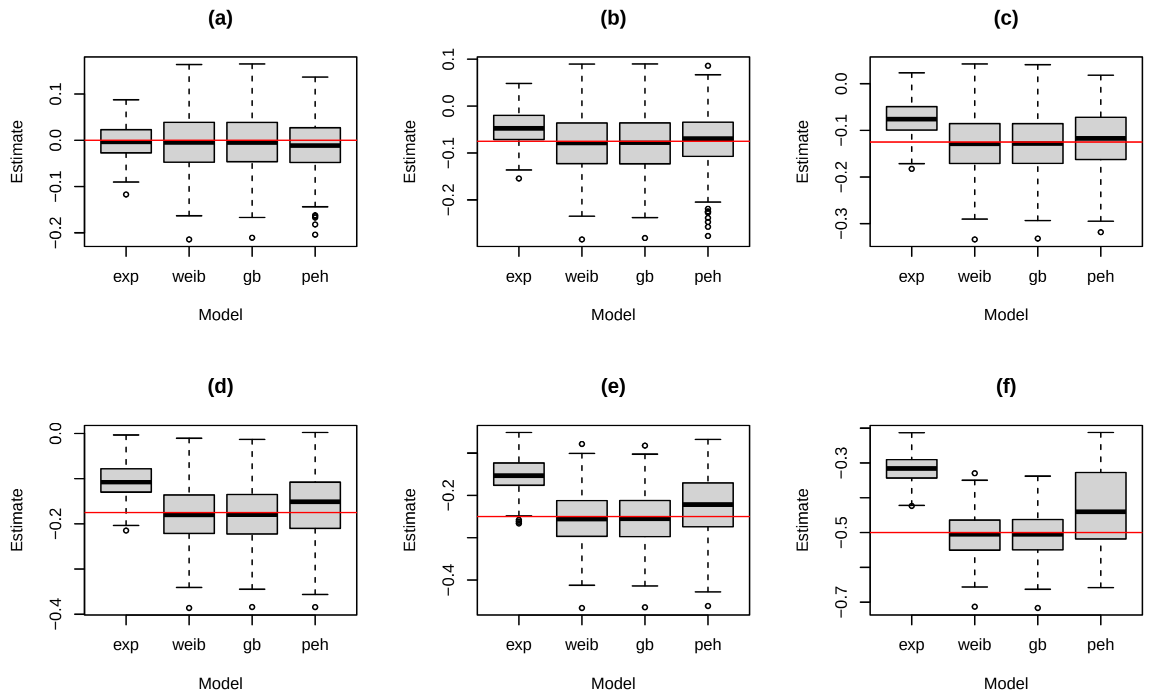

Figure SM.5: Distribution of posterior modes for the treatment effect from trial simulation with a fixed sample size when data were generated from the Weibull model with (a) $\beta = 0$, (b) $\beta = -0.075$, (c) $\beta = -0.125$, (d) $\beta = -0.175$, (e) $\beta = -0.25$, (f) $\beta = -0.5$ and the treatment effect was estimated based on the exponential ('exp'), Weibull ('weib'), general Bayesian ('gb') and piecewise exponential hazard ('peh') models. All results are based on 500 trial simulations.

4.2 Distribution of posterior modes for the treatment effects from trial simulation with an adaptive design

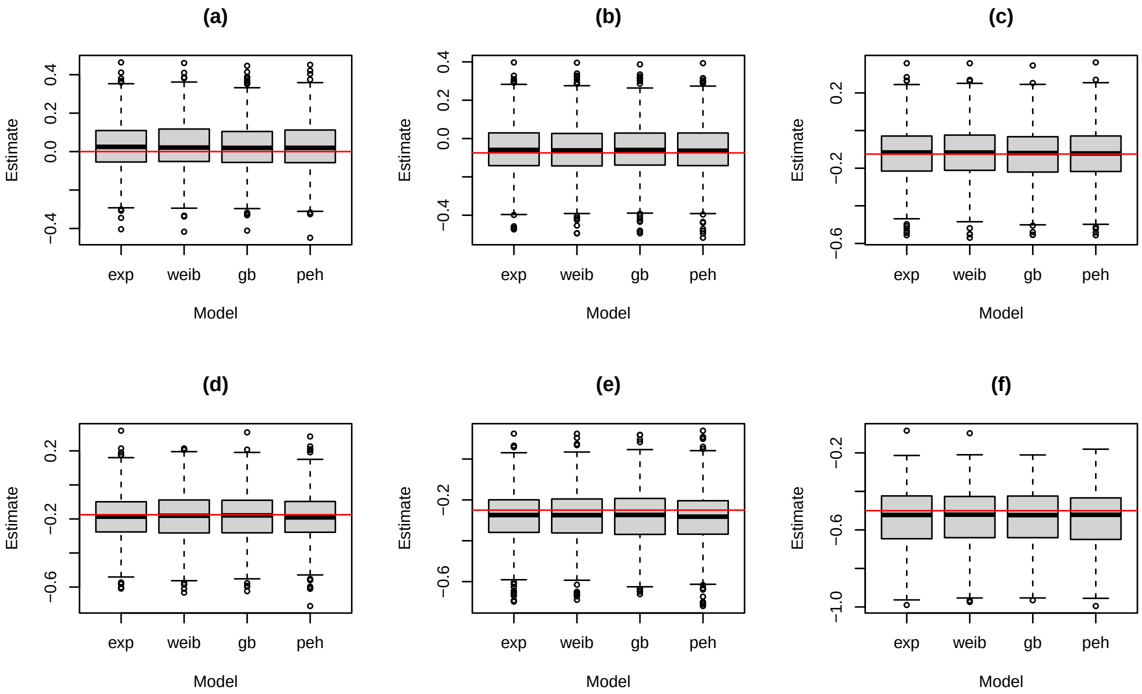

Figure SM.6: Distribution of posterior modes for the treatment effect from trial simulation with an adaptive design when data were generated from the exponential model with (a) $\beta = 0$, (b) $\beta = -0.075$, (c) $\beta = -0.125$, (d) $\beta = -0.175$, (e) $\beta = -0.25$, (f) $\beta = -0.5$ and the treatment effect was estimated based on the exponential ('exp'), Weibull ('weib'), general Bayesian ('gb') and piecewise exponential hazard ('peh') models. All results are based on 500 trial simulations.

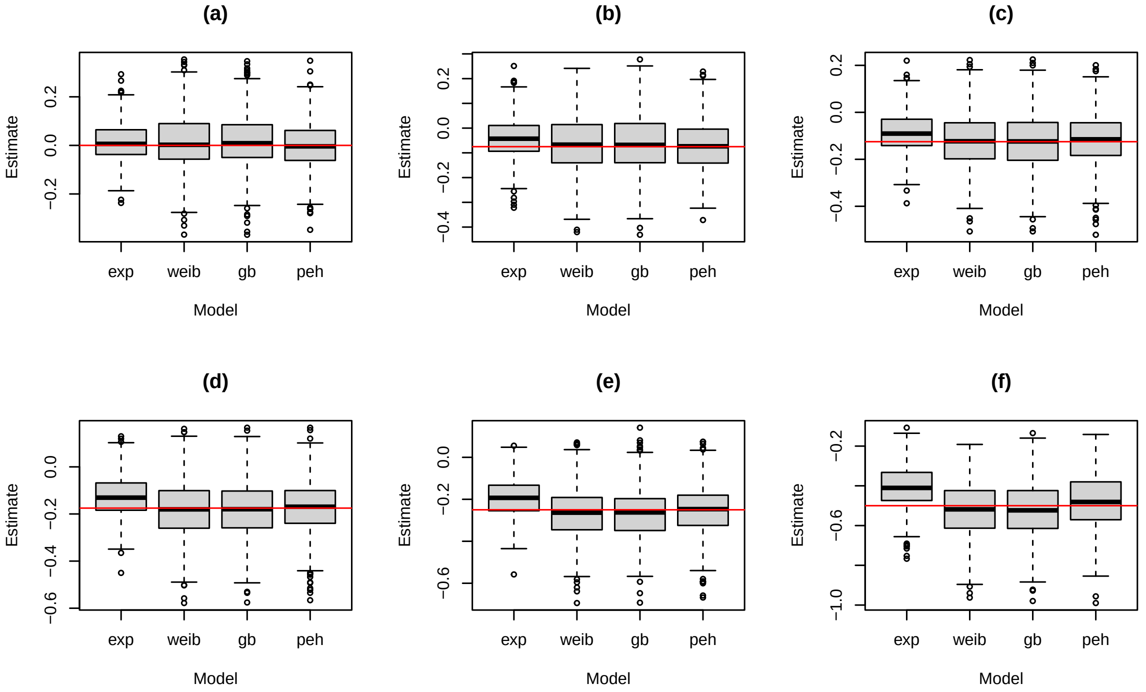

Figure SM.7: Distribution of posterior modes for the treatment effect from trial simulation with an adaptive design when data were generated from the Weibull model with (a) $\beta = 0$, (b) $\beta = -0.075$, (c) $\beta = -0.125$, (d) $\beta = -0.175$, (e) $\beta = -0.25$, (f) $\beta = -0.5$ and the treatment effect was estimated based on the exponential ('exp'), Weibull ('weib'), general Bayesian ('gb') and piecewise exponential hazard ('peh') models. All results are based on 500 trial simulations.

5 Additional results discussed in Section 8 of the paper

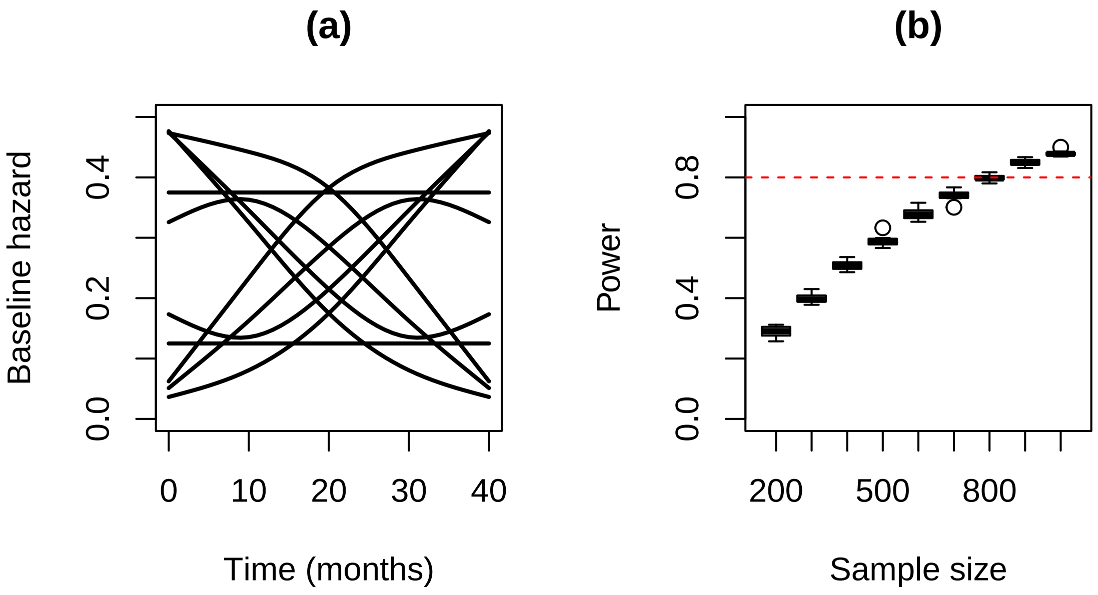

Figure SM.8: (a) Set of 10 baseline hazard functions that could be observed under the super model. (b) Distribution of the results from the power analysis for the illustrative example under the set of 10 baseline hazard models given in (a). All results are based on 500 trial simulations.

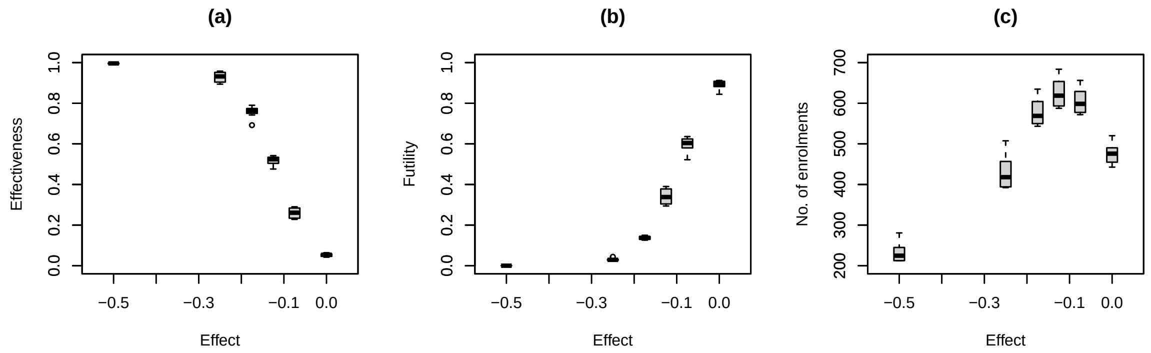

Figure SM.9: For the ORVAC trial, the distribution of the proportion of trials stopped due to (a) effectiveness or (b) futility with the (c) associated distributions for the average number of enrolments when the data were generated from 10 baseline hazard functions (see Figure SM.8(a)) that could be observed under the super model.
